# Case Report: CD19 CAR-T cells derived from recipient of umbilical cord blood transplantation effectively treated relapsed acute lymphoblastic leukemia after UCBT

**DOI:** 10.3389/fimmu.2025.1586349

**Published:** 2025-05-19

**Authors:** Hua Li, Xiaofan Li, Na Xian, Gangxiong Huang, Nainong Li

**Affiliations:** ^1^ Department of Hematology, Xiamen Medical College Affiliated Second Hospital, Xiamen, China; ^2^ Hematopoietic Stem Cell Transplantation Center, Fujian Institute of Hematology, Fujian Provincial Key Laboratory on Hematology, Department of Hematology, Fujian Medical University Union Hospital, Fuzhou, China; ^3^ Institute of Immunotherapy, Fujian Medical University, Fuzhou, China; ^4^ Tcelltech Biological Science and Technology Inc., Fuzhou, China

**Keywords:** chimeric antigen receptor T (CAR-T), relapsed acute lymphoblastic leukemia, allogeneic umbilical cord blood transplantation (UCBT), Ph+ acute lymphoblastic leukemia, umbilical cord derived CART

## Abstract

Recent advances in chimeric antigen receptors have provided an alternative approach for treating relapsed acute lymphocyte leukemia after allogeneic hematopoietic stem cell transplantation (allo-HSCT). However, relapsed patients who had undergone allogeneic umbilical cord blood transplantation (UCBT) have no chance of having CAR-T cells derived from donors due to lacking UCB. We present a case of a patient with Ph+ ALL who relapsed after UCBT and achieved complete morphological and molecular remission following treatment with CD19 CAR-T cells derived from the recipient post-UCBT. The patient had only grade I CRS. GVHD or neurotoxicity was not observed. More than 6 years after CAR-T cell infusion, the patient was still in hematologic and molecular complete remission with negative minimal residual disease (MRD). This case is the first to show a new strategy of practicality, efficacy, and safety of CD19 CAR-T cells derived from UCBT recipients for treating relapsed ALL after UCBT.

## Background

**Figure 1:**
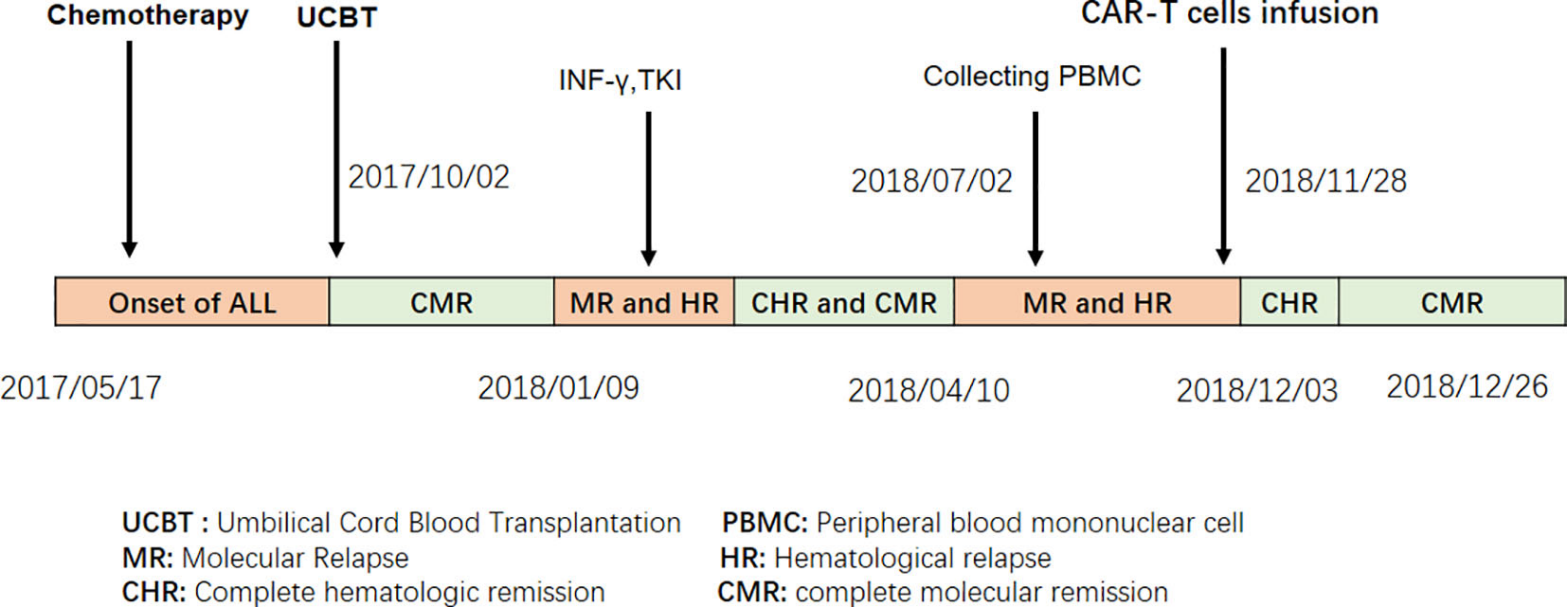
Treatment procedures for the ALL patient who, after UCBT, received autologous CAR T cell therapy due to relapse.

Adult patients with acute B-cell lymphoblastic leukemia (B-ALL) often relapse after chemotherapy alone, with a long-term survival rate of approximately 30% (1, 2). Although hematopoietic stem cell transplantation (HSCT) has improved the survival of patients with hematologic malignancies, relapse after HSCT remains a challenge. Progressive malignancy is the leading cause of death following allo-HSCT (3). Patients with relapsed acute lymphoblastic leukemia after allo-HSCT have a median survival of 5.5 months (4). Donor leukocyte infusion (DLI) is commonly used for patients with high-risk relapse post-HSCT, but its efficacy is 15%–40% and induces severe graft-versus-host disease (GVHD), which increases transplantation-related mortality (5, 6). Recent advances in chimeric antigen receptor (CAR) T-cell therapy have shown significant progress and have changed the landscape of treatment for hematologic malignancies (7). Multiple clinical trials have demonstrated that infusion of CD19 CAR-T cells resulted in overall remission rates of 70%–90% in patients with relapsed B-cell ALL after autologous and allogeneic donors (8–11). However, relapsed patients who received UCBT do not have a chance for DLI or CAR-T cells from donors owing to the limitation of the source. To solve this problem, we attempted a new strategy of CAR-T cell therapy in UCBT recipients. We report that a patient with B-ALL who relapsed after UCBT achieved a second complete remission after treatment with CD19 CAR-T cells derived from the recipient (**Figure 1**).

## Case representation

A 27-year-old man presented to our hospital with cough and fever in May 2017. Peripheral blood examination revealed a white blood cell count of 183.5 × 10^9^/L, hemoglobin level of 110 g/L, and platelet count of 119 × 10^9^/L. Bone marrow examination revealed 88% blast cells with negative myeloperoxidase staining. Flow cytometry analysis revealed an abnormal blast population (48%) expressing HLA-DR, CD10, CD19, CD22, CD33, CD34, CD38, CD58, CD123, cCD79a, and TdT. Cytogenetic and molecular biology analyses revealed t (9, 22)(q34;q11) and BCR-ABL1(p210) fusion gene transcripts. No other gene mutations were detected by next-generation DNA sequencing. The patient was diagnosed with Philadelphia chromosome-positive B-cell acute lymphoblastic leukemia (Ph^+^ B-ALL).

## Treatment

Reducing tumor load with cyclophosphamide and prednisone was given to, following induction chemotherapy with Vindesine, Idarubicin, Cyclophosphamide, Prednisone, and L-asparaginase (VICLP). He received one more cycle of VICLP and started a tyrosine-kinase inhibitor (imatinib) for consolidation. Complete hematologic remission (CHR) was achieved for two months. Subsequently, on 2 October 2017, the patient received a single allogeneic unit of UCBT with a myeloablative regimen of busulfan/cyclophosphamide. Peripheral myeloid engraftment (absolute neutrophil count, >0.5 ×10 ^9^/L) was evident on day 22, and he was platelet transfusion-dependent (platelet count, >20 × 10^9^/L) until day 50 post-transplantation. Donor HLA-matched complete chimerism (100%) was achieved within the initial 14 days. The patient achieved a complete molecular remission (CMR) 3 months after transplantation. At molecular relapse, the patient was treated with interferon-γ for its antitumor effects and received BCR-ABL-targeted therapy using the TKI Dasatinib.

**Figure 2:**
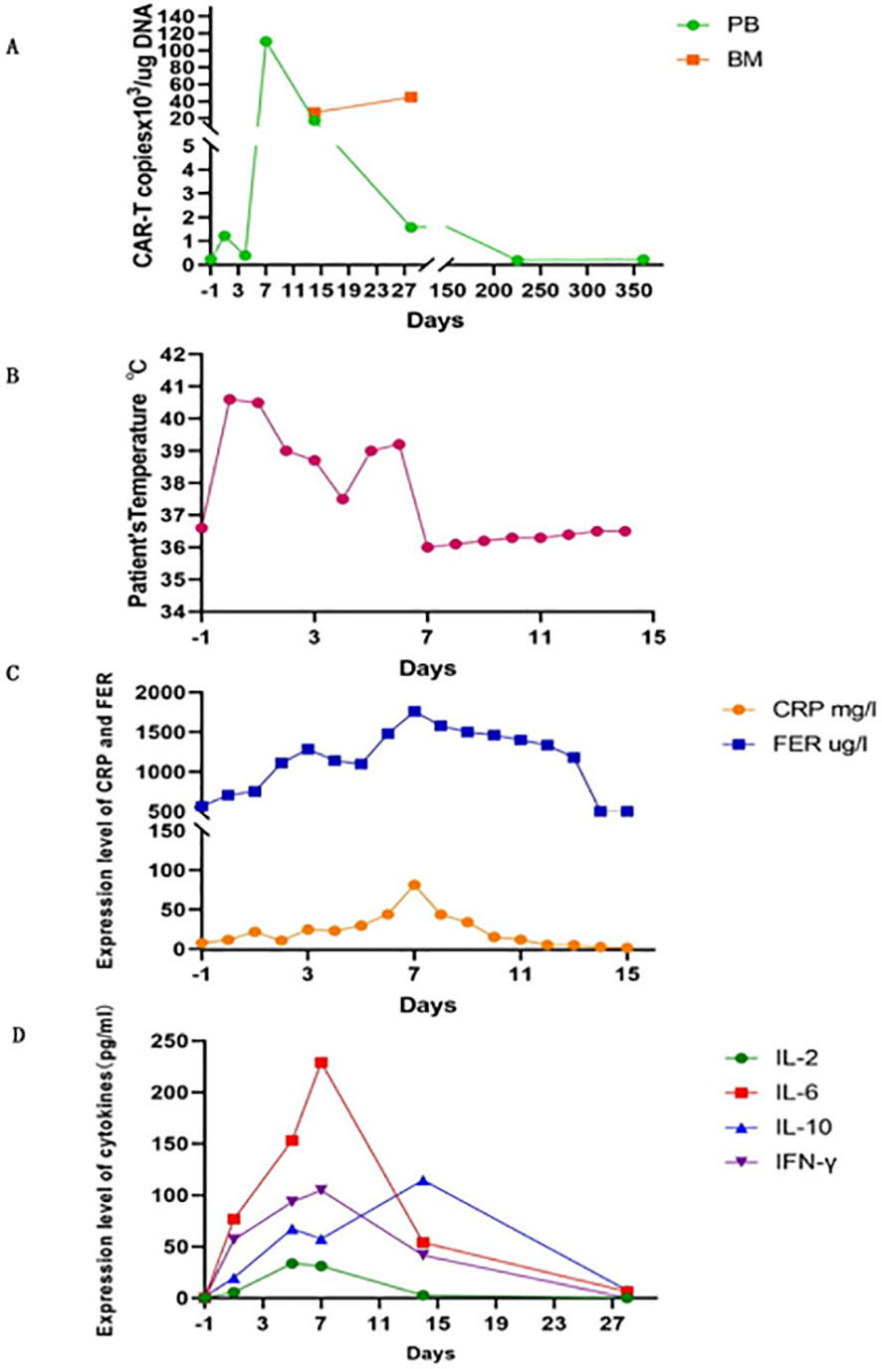
CAR-T expansion *in vivo*, patient’s temperature, CRP and ferritin, serum levels of cytokines after CAR-T cell infusion. **(A)** The number of CAR-T cells expansion in PB and BM. **(B)** Patient’s temperature. **(C)** Changes in CRP and ferritin. **(D)** Serum levels of cytokines. PB, peripheral blood; BM, bone marrow.

After treatment, the patient achieved complete CHR with sustained MRD negativity, as confirmed using flow cytometry. However, molecular monitoring revealed persistent detection of the BCR-ABL1 fusion gene by reverse transcription-polymerase chain reaction (RT-PCR) for 2 months. But the disease was going to progress over the next 3 months. CAR-T cell therapy was performed after obtaining informed consent from the patient. When the patient relapsed with 7% blast cells in the bone marrow and BCR-ABL1 30.88% (IS), analysis of short tandem repeats (STRs) showed 100% chimerism with umbilical cord donor cells. Peripheral blood mononuclear cells (PBMC) from the patients were collected for preparation of CAR-T cells. Following isolation and procurement, T-cells were *ex vivo* activated and transduced using a lentiviral vector encoding the CAR gene. CD19-targeting CAR-T cells were generated using a murine single-chain antibody with a 4-1BB co-stimulatory domain carrying IL-6shRNA. After continuous *in vitro* culture for 7 days–10 days, testing was conducted in accordance with the relevant standards to ensure the function and safety of the final product, including the quantification of target CD19 CAR-T cells, bacteria, mycoplasma, endotoxins, and other potential contaminants. The patient then received a conditioning regimen of fludarabine (30 mg/m^2^) and cyclophosphamide (300 mg/m^2^) from Days-4 to -2 before CAR-T cell therapy. Before CAR-T cell infusion, the patient had 9.5% leukemia blast cells in the bone marrow. STR analysis decreased to 93.66%. On 8 November 2018, 7.8 × 10^6^/kg of CD19 CAR-T cells were transfused on day 0. The number of CAR-T cells reached a peak on the 7th day after infusion, which expanded to 110 folds *in vivo* (**Figure 2A**; **Table 1**).

**Table 1:** The number of CAR-T cells expansion in PB and BM.

Days post CAR-T cells infusion	Peripheral blood (copies x10^3^/ug)	Bone marrow (copies/ug)
D1	1.22	/
D4	0.39	/
D7	110.63	/
D14	17.09	26.73
D28	1.58	44.87
D120	2.15	/
D225	0.19	/
D360	0.23	/

## Outcome and follow-up

The patient developed high fever for 1 week and diarrhea for 5 days after CAR-T cell infusion (**Figure 2B**). The patient’s temperature tended to be normal, and diarrhea was relieved after treatment with nonsteroidal drugs (indomethacin) and supportive care. The levels of cytokines, including plasma interleukin IL-6, IL-2, IL-10, interferon-γ, C-reactive protein (CRP), and ferritin, significantly increased with CAR-T cell expansion (**Figures 2C, D**). IL-6 levels peaked at 229 pg/ml (200-fold higher than the baseline) on day 7. Grade I cytokine release syndrome (CRS) was diagnosed. No hypotension, tachycardia, hypoxia, coagulopathy, or multiple organ failure was observed. GVHD did not occur. On day 30 after CAR-T cell infusion, the patient regained CHR and CMR with negative BCR-ABL. After 6 years, the patient underwent annual tests, including routine blood tests, bone marrow cytology, MRD, and quantitative detection of the BCR::ABL1 P210 fusion gene and STR. The patient remained in hematological and molecular complete remission.

## Discussion

Relapse after chemotherapy is the main cause of death in patients with ALL, as well as in patients after allo-HSCT. Donor lymphocyte infusions (DLIs) and allogeneic anti-CD19 CAR-T cells from HLA-matched donors can effectively treat progressive B-cell malignancies. However, DLI and allogeneic donor-derived CAR-T cells are not available for UCBT recipients. Autologous and allogeneic CD19 CAR-T cells have achieved complete remission in previously treated relapsed/refractory B cell malignancies (10, 12–14). In our case, therapy with CAR-T cells derived from UCBT recipient-self was the only strategy for treating relapse after UCBT. When T cells were collected to generate CAR-T cells, chimerism in the UCBT patient was 100%. The patient experienced CR again, with a negative MRD after CAR-T cell therapy. Our case is the first to show the feasibility, safety, and efficacy of CD19 CAR-T cells derived from a recipient of UCBT for treating relapsed Ph^+^-ALL. This strategy is important for patients who have no DLI treatment after UCBT. This provides an alternative approach to overcome the barriers of UCBT recipients who have no better treatment choices for relapsed ALL.

The current duration of complete remission in our patient was more than 6 years after CAR-T cell infusion. CAR-T cells were still detected 12 months after infusion. Park et al. (15) found that a higher ratio of peak CAR T-cell expansion to tumor burden significantly correlated with the event-free survival and overall survival, which was a better predictor of long-term survival. In terms of the efficacy of CAR-T cell therapy, the ratio of infused CAR-T cell expansion fold to tumor load was superior to the absolute magnitude of T-cell expansion in patients receiving CAR-T cell treatment. Clinical trials have demonstrated that persistence of CAR-T cells is a key factor in the success of CD-19 CAR-T cell therapy (16). The 4-1BB co-stimulator plays the role prolonged CAR-T cell persistence. It was reported that 4-1BB based CAR-T cells in the blood can persist for a median duration of 168 days (range, 20 days–617 days) (17). In our case, the number of infused CAR-T cells was expanded to 110 folds and sustained for 12 months in the recipient. Therefore, we believe that CAR-T cell expansion folds and CAR-T cell persistence of 4-1BB co-stimulation contributed to the efficacy of CAR-T cell therapy in our case. Additionally, some studies have indicated that umbilical cord-derived CAR-T therapy exhibits favorable clinical outcomes. Xu et al. reported that 11 patients with R/R B-ALL after UCBT following CD19-targeted CAR-T therapy achieved a high remission rate and experienced mild adverse events (18). Marra et al. report that a patient with multiple relapsing Ph+ B-ALL achieved good clinical outcomes after CAR-T therapy after UCBT. The case report also confirmed the positive therapeutic effects of umbilical cord-derived CAR-T therapy (19). Notably, in our case, the patient received CAR-T therapy after UCBT and achieved long-term survival for more than 6 years, which may have contributed to the patient’s sustained remission. First, at the time of peripheral blood T-cell collection, the patient’s bone marrow blasts were only 7%, indicating a low tumor burden at relapse. Second, short tandem repeat (STR) analysis confirmed 100% cord blood chimerism. CD19 CAR-T cells derived from recipient T-cells after UCBT. This represents CAR-T cells derived from cord blood (20–22), which exhibit characteristics of cord blood-derived T cells. This contributes to a robust graft-versus-leukemia (GVL) effect, similar to that of fresh cord blood-derived T cells, thereby potentially enhancing the therapeutic efficacy in this clinical setting.

CRS is a common side effect of immune-mediated response to CAR-T cell therapy. In our case, the patient developed grade I CRS with fever and diarrhea according to the revised grading system (23). The cytokines detected included IL-6, IL-2, IL-10, and IFN-γ, which increased dramatically on the 7th day after CAR-T cell infusion. They coincided with CD19 CAR-T cell expansion *in vivo* and then decreased rapidly to the normal range within one month. The peaks of CRP and ferritin lagged behind the tested cytokine levels by 1 to 2 days. CRS is caused by the release of numerous cytokines from CAR-T cells and other cells including monocytes, macrophages, and dendritic cells, which generally occurs 1 to 14 days after CAR-T cell infusion and is sustainable for 1–10 days (24–27). Risk factors for CRS include tumor load, the time of CAR-T cell infusion, infection status, the amount of CAR-T cells for infusion, and the preparative regimen of CAR-T cell therapy (15, 28, 29). Patients with a low tumor burden have a markedly lower incidence of CRS and neurotoxic events (15). The patient’s CRS with fever and diarrhea was grade I and was managed well. We believe that the efficacy of CAR-T cell therapy in this case might be due to the lower tumor load before CAR-T cell therapy and the infusion of an appropriate number of CAR-T cells. Furthermore, upon observing the clinical and laboratory findings in the patient, we promptly initiated supportive measures and nonsteroidal drug (indomethacin) intervention to prevent subsequent severe CRS events. Finally, compared to CD28 as a costimulatory domain, CAR-T cells with a 4-1BB costimulatory domain induce less severe CRS events (30). Based on the data presented, it is evident that the incidence of severe cytokine release syndrome (CRS) associated with CAR-T cell treatment is remarkably low.

In the present case, GVHD and neurotoxicity were not observed. Our patient exhibited 100% chimerism after undergoing UCBT. Although the infusion of CAR-T cells derived from patients post-UCBT was alloidentical transplantation (31), we believe that the CD19 CAR-T cells made from the recipient of UCBT would cause no GVHD or less GVHD, and that the transplanted grafts in UCBT recipients would not be attacked by their own CAR-T cells. The levels of acute and chronic GVHD after infusion of recipient-derived CAR-T cells were lower than those of allo-derived CAR-T cells. In this case, it is hypothesized that the recipient’s tolerance to the cells facilitates avoidance of allo-rejection (32). We believe that the lack of GVHD in this patient may be due to the following reasons: First, the lower dose of CAR-T cells reduced the possibility of GVHD compared with DLI. Second, T cells from a patient after UCBT may have been tolerated by the recipient’s immune system. CAR-T cell therapy has shown better clinical efficacy in patients with R/R B-ALL after UCBT. CAR-T cells of umbilical origin may facilitate rapid expansion and enhance the therapeutic effect of the treatment. Therefore, further confirmation with a larger sample size is needed to validate these findings.

## Data Availability

The original contributions presented in the study are included in the article, further inquiries can be directed to the corresponding authors.
